# The efficacy and safety of an indocyanine green−hyaluronic acid mixture (LuminoMark™) for localization in patients with non-palpable breast lesions: A multicenter, randomized, open-label, parallel phase 3 clinical trial

**DOI:** 10.3389/fonc.2023.1039670

**Published:** 2023-03-24

**Authors:** Yoon Ju Bang, Hee Jun Choi, Isaac Kim, Moo-Hyun Lee, Seeyoun Lee, Hyuk Jai Shin, Seok Jin Nam, Jeong Eon Lee, Byung-Joo Chae, Se Kyung Lee, Jai Min Ryu, Seok Won Kim

**Affiliations:** ^1^ Department of Surgery, Samsung Changwon Hospital, School of Medicine, Sungkyunkwan University, Changwon, Republic of Korea; ^2^ Department of Surgery, CHA Bundang Medical Center, CHA University, Seongnam, Republic of Korea; ^3^ Department of Surgery, School of Medicine, Keimyung University, Dongsan Medical Center, Daegu, Republic of Korea; ^4^ Center for Breast Cancer, National Cancer Center Hospital, Goyang, Republic of Korea; ^5^ Department of Surgery, Myongji Hospital, College of Medicine, Hanyang University, Goyang, Republic of Korea; ^6^ Division of Breast Surgery, Department of Surgery, Samsung Medical Center, School of Medicine, Sungkyunkwan University, Seoul, Republic of Korea

**Keywords:** localization, non-palpable, excision, indocyanine green, indocyanine green (ICG)

## Abstract

**Purpose:**

The incidence of early tumor detection is increasing due to popularization of breast cancer screening and the development of imaging techniques. Thus, suitable preoperative localization is required for proper diagnosis and treatment of non-palpable breast lesions. The purpose of this study was to evaluate the efficacy and safety of indocyanine green (ICG)-hyaluronic acid (HA) mixture for lesion localization compared to activated charcoal.

**Methods:**

This was a multicenter, randomized, open-label, parallel phase 3 clinical trial performed at four centers in Korea. Female patients scheduled for surgery to remove non-palpable breast lesions were enrolled. One hundred and nine patients were randomly assigned to a control group (activated charcoal: 0.3. – 1 mL) or a study group (ICG-HA mixture, 0.2 mL) for the localization of a breast lesion. The primary endpoint was the accuracy of resection. Secondary endpoints included the technical success rate, histopathological accuracy, skin pigmentation rate, and adverse event rate.

**Results:**

A total of 104 patients were eligible for per-protocol analysis (control group, n = 51; study group, n = 53). The accuracy of resection in the study group was not inferior to that of the control group (90.57% vs. 98.04%, 95% confidence interval (CI): -2.31 – 18.91, *p* = 0.21). There was no statistically significant difference in technical success rate between the two groups (marking on breast skin: *p* = 0.11, marking on the excised specimen: *p* = 0.12). However, there were statistically significant differences in histopathological accuracy (0.26 ± 0.13 vs. 0.33 ± 0.17, *p* = 0.01) and skin pigmentation rate (0.00% vs. 30.77%, *p*< 0.01). Adverse events were not reported in either group.

**Conclusions:**

When localization was performed using ICG-HA, the accuracy of resection was not inferior to that of activated charcoal. However, skin pigmentation rate was significantly lower. In conclusion, ICG-HA is effective and safe for localizing of non-palpable breast lesions.

## Introduction

The detection of early tumor which is non-palpable and can only be found using precise localization has increased with the popularization of breast ultrasound (US) screening and the advent of imaging techniques. Preoperative localization for non-palpable breast lesions is very important for minimal but accurate excision of non-palpable breast lesions for proper diagnosis and treatment (1). Various procedures, including injection of a bioavailable dye, wire localization, radioactive seed localization and skin marking with an oil-based or water-based pen to pre-mark the non-palpable lesion, have been used so that lesions could be distinguished during surgeries (2–5).

Wire localization is a classic and widely used technique with several disadvantages. It should be performed on the day of surgery because of its disadvantages such as risk of wire migration or withdrawal, pain to patients, and interference with surgical approaches (2, 6, 7). US-guided localization using bioavailable dye for visualizing non-palpable breast lesions is rapid and easy to perform without using mammography (MMG). It has been shown to decrease positive cancer margin rates and re-operation rates (8). Charcoal tattooing is a widely used method without the risk of fast dye dispersion. Surgery can be planned after a few days. However, it has disadvantages of skin pigmentation and foreign body reaction (9).

Indocyanine green (ICG) approved by Food and Drug Administration (FDA) is the most widely accepted fluorophore used in various clinical fields, including sentinel lymph node (SLN) mapping, identification of solid tumors, lymphography, angiography, and anatomical imaging during surgery (10–16). In the field of breast oncology, ICG has been used for monitoring skin perfusion in nipple-sparing mastectomies to guide the locations of mastectomy incisions and minimize ischemic complications, and for performing sentinel lymph node biopsy (17). However, there is no report of tattooing localization using ICG under US-guidance for non-palpable breast lesions. Through a phase-2 clinical trial, we have suggested that ICG-hyaluronic acid (HA) (LuminoMark™) can be used for accurate preoperative localization without skin pigmentation in benign breast diseases (18). The aim of this study was to evaluate the efficacy and safety of ICG-HA for localizing non-palpable breast lesions including breast cancer and confirm that ICG-HA is not inferior to activated charcoal, which has been widely used.

## Materials and methods

### Study design

This was a multicenter, randomized, open-label, parallel phase 3 clinical trial done at four centers, Samsung Medical Center, Dongsan Medical Center, National Cancer Center, and Myongji Hospital in Korea (ClinicalTrials.gov Identifier: NCT 04606329). This study aimed to evaluate the efficacy and safety of ICG-HA as a novel mixture for localization compared to activated charcoal.

**Figure 1:**
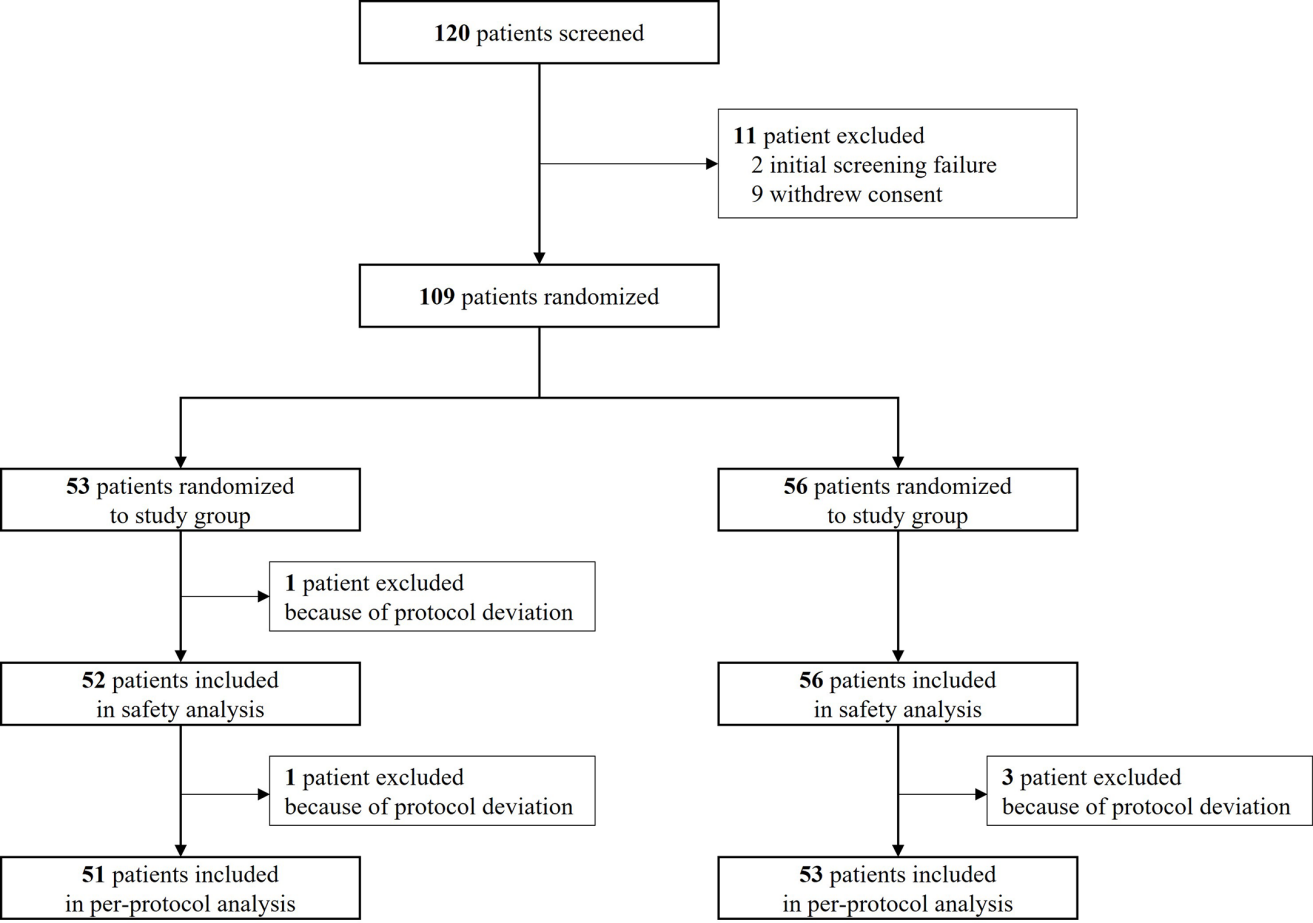
The study flow.

This clinical trial was designed to last for eight weeks, including screening, randomization, localization, surgery, and follow-up visits. **Figure 1** shows the study flow chart. After randomization, localization was performed at visit 2 or 3. At visit 3, surgery and the first evaluation of efficacy and safety were performed. Efficacy and safety were evaluated again at visits 4 and 5.

To calculate the number of subjects to prove the non-inferiority of ICG-HA to activated charcoal, the negative resection margin rate of the study group and the control group was set at 81.1% (9). The limit of non-inferiority was set at 22.5% by referring to a previous study (19) that compared localization methods in patients who underwent surgery to remove non-palpable breast lesions. A sample size of 54 in each group achieved a power of 80% to detect a difference between group proportions of -0.225.


$$\text{N}=\frac{{\left[z_{1-\alpha }\times \sqrt{p_{s}\left(1-p_{s}\right)+p_{c}\left(1-p_{c}\right)}+z_{1-\beta }\times \sqrt{p_{s}\left(1-p_{s}\right)+p_{c}\left(1-p_{c}\right)}\right]}^{2}}{{\left(p_{s}-p_{c}-\delta \right)}^{2}}$$

$$=\frac{{\left[{\text{z}}_{0.975}\times \sqrt{2\times 0.811(1-0.811)}+{\text{z}}_{0.8}\times \sqrt{2\times 0.811(1-0.811)}\right]}^{2}}{{\left(-0.225\right)}^{2}}=47.53\approx 48$$

1) Level of significance, α= 0.025 (one-sided)

2) Power of the test, 1-β= 0.8

3) Allocation ratio= 1:1

4) Resection margin negative rate; study group, *p_t_* 0.811

5) Resection margin negative rate; control group, *p_c_* 0.811

6) Limit of non-inferiority, -δ= -0.225

In this clinical trial, 0.2 mL of ICG-HA mixture (LuminoMark™, Hanlim Pharm. Co., Ltd., Korea) injection was set as the dose of the test drug, and 0.3 – 1 mL of activated charcoal (Chacotrace^®^, Phebra, Australia) was set as the control according to the results of the phase 2 clinical trial (18).

### Patients and procedures

The study population targeted females aged between 19 and 80 years who were scheduled for surgeries to remove non-palpable breast lesions confirmed by breast US, regardless of the type of lesion. Palpability of the breast lesion was evaluated by the surgeon at the first visit. There was no limit to the size of the lesion since palpability depended not only on the size of the lesion, but also on the breast size and the location of the lesion. Inclusion and exclusion criteria are detailed in **Table 1**.

**Table 1:** Inclusion and exclusion criteria.

Inclusion Criteria
Patients aged 19 to 80 years
Patients with a non-palpable breast lesion confirmed by mammography or breast ultrasonography
Those who were scheduled to undergo surgery to remove breast lesions
Patients who provided informed consent to participate in the study
Exclusion Criteria
Patients who were scheduled to undergo total mastectomy without requiring localization
Patients with multiple tumors or extensive microcalcifications
Patients with positive margins despite having undergone local resection three or more times
Patients who had received neoadjuvant chemotherapy
Patients with active connective tissue disease (scleroderma or lupus, etc.) that invaded the skin
Patients with locally advanced breast cancer or inflammatory breast cancer
Patients with a history of hypersensitivity to the main ingredients or drug excipients
Patients who did not agree to use contraception during the clinical trial period
Pregnant or lactating women
Patients taking part in other clinical trials
Patients with any condition (social or medical) that, in the opinion of the investigator, would make study participation unsafe

**Figure 2:**
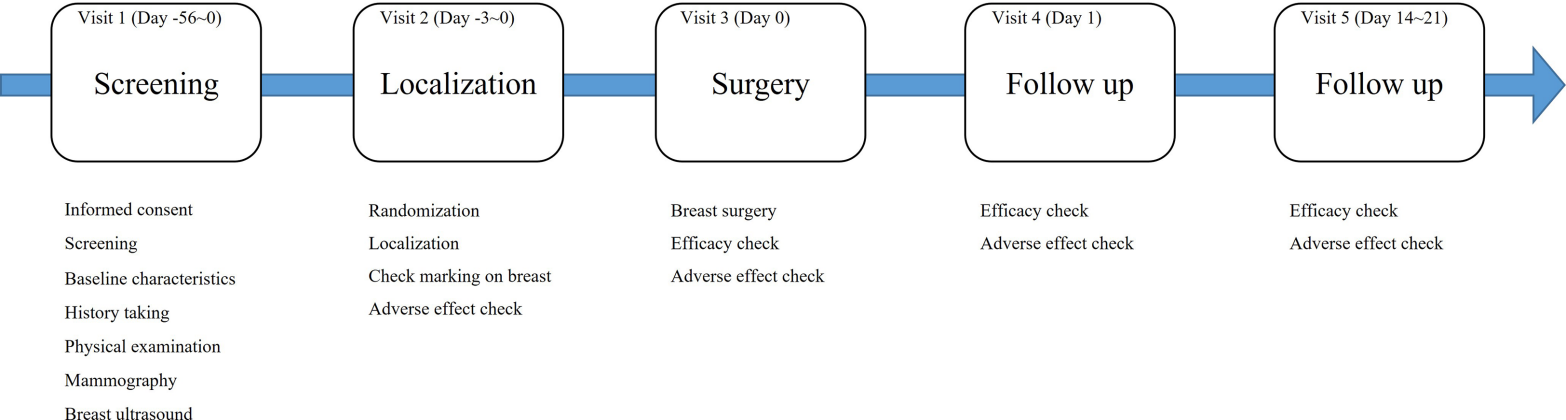
Flow chart showing participant enrollment.

**Figure 2** shows a flow chart of participant enrollment. A total of 120 provided consent to participate in this clinical trial. However, 11 patients were excluded after screening. Thus, 109 patients were randomized at a 1:1 ratio to a control group (N = 56) and a study group (N = 53). Among these 109 randomized patients, 108 (52 in the study group and 56 in the control group) were included in the safety analysis after excluding one patient who missed injection of the clinical trial drug. One hundred and six patients (51 in the test group and 55 in the control group) in the safety analysis (SA) group were included in the full analysis (FA) group after excluding two patients with missing resection margin evaluations. Of these 106 patients in the FA group, 104 patients (51 in the test group, 53 in the control group) were included in the per-protocol (PP) analysis after excluding two patients for violating the inclusion/exclusion criteria and measurement timing of the primary efficacy endpoint.

**Figure 3:**
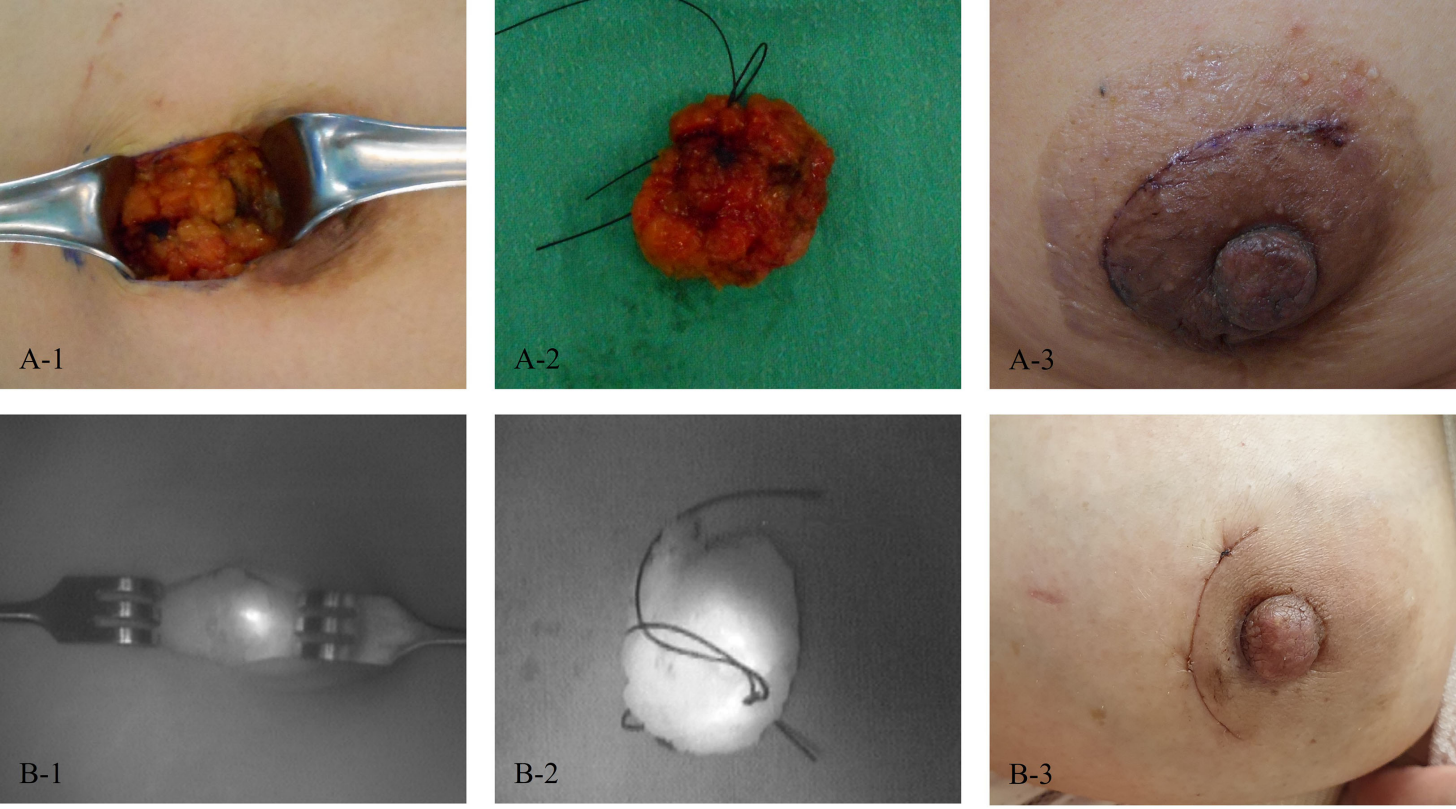
Photos of patients. **(A)** A patient in the control group (1, After skin incision; 2, After excision; and 3, On the last follow-up day). **(B)** A patient in the study group (1, After skin incision; 2, After excision; and 3, On the last follow-up day).

After randomization, localization was performed for the target lesion by injecting 0.2 mL of Luminomak™ (study group) using a 26-gauge needle or 0.3 – 1 mL of Chacotrace^®^ (control group) using a 18-gauge needle. Skin excision to avoid skin pigmentation was not allowed. However, it was allowed if skin excision was necessary for complete removal because the lesion was close to the skin. In the case of multiple lesions, one target lesion was selected by the investigator and the test drug was used only for the target lesion. Other lesions were also removed according to the general method of each institution. Localization with ICG-HA was visualized using near-infrared fluorescence. Intraoperative photographs were taken after skin incision and excision. Follow-up photographs were then obtained (**Figure 3**).

### Evaluation criteria

The primary endpoints were accuracy of resection determined as the proportion of patients who had negative resection margins and histopathological accuracy. As assessed by the pathologist, a negative margin was defined as absence of the target lesion in any resected section of the breast specimen. Although additional resection was performed according to frozen biopsy results and a negative margin was finally obtained during breast cancer surgery, margin status was defined based on initial frozen biopsy results. Histopathological accuracy was defined as the longest length of the breast lesion compared to the longest length of the resected specimen for evaluating whether the target lesion was accurately excised without unnecessary wide resection. Secondary endpoints included the technical success rate, skin pigmentation rate, and adverse event rate. The technical success rate was defined as successful visualization after localization. It was evaluated for breast surface before surgery and excised specimen, respectively. Skin pigmentation was defined as discoloration due to the drug. It was evaluated by photographing the surgical site after surgery. The incidence of adverse events was defined as the proportion of patients with any confirmed adverse events after surgery. All events were collected including any harmful and unintended signs, symptoms, abnormalities in clinical laboratory test results, and diseases that occurred to the patients after injection of the test drug. In this clinical trial, postoperative pain, nausea, and vomiting were not collected because surgery was performed. The investigator evaluated whether the reported event was related to the test drug. Any events evaluated other than ‘definitely not related’ were defined as adverse events.

### Statistical analysis

Primary efficacy analysis was based on Chan and Zhang’s 95% (two-sided) confidence interval (CI) for the difference in negative resection margin between the two groups. Non-inferiority of the study group compared to the control group was established if the difference in the negative resection margin rate was greater than the lower non-inferiority margin, i.e., if the lower boundary of the two-sided 95% CI was greater than or equal to -22.5%. All other statistical significance tests were performed as two-sided tests with a 5% significance level. Statistical significance was considered when *p*-values of< 0.05. Categorical data in this study were analyzed for differences using chi-squared test or Fisher’s exact test. In addition, McNemar’s test was used to analyze whether there was any change within the group. Continuous data in this study were analyzed for differences using paired t-test or Wilcoxon’s signed-rank test. All statistical analyses were performed using SAS software (SAS Institute Inc, version 9.4). Since results for the PP population were the same as those for the FA population, only PP data are presented in this paper. Safety population included all patients receiving at least one dose of the study drug.

### Ethical statement

This clinical trial was conducted in accordance with the protocol approved by the Ministry of Food and Drug Safety (MFDS) and the Institutional Review Board (IRB) for each institution. It complied with Korea’s Good Clinical Practice (KGCP) and Good Clinical Practice (GCP) set forth by the International Conference on Harmonisation (ICH).

We declare that this study has obtained a report of ethics board approval. Written informed consent was obtained from each participant. This study was approved by the Ethics Committee of Samsung Medical Center (IRB No. SMC 2019-12-117), Dongsan Medical Center (IRB No. DSMC 2020-01-047), National Cancer Center (IRB No. NCC2020-0070), and Myongji Hospital (IRB No. MJH2020-01-011).

## Results

A total of 104 patients were eligible for PP analysis (control group, n = 51; study group, n = 53). **Table 2** shows patient characteristics. There were no significant differences in baseline characteristics between the two groups. Each dye was administered within 3 days of surgery. There was no difference in the mean exposure periods between the two groups (p = 0.86). The size of the lesion measured by preoperative US varied from 0.1 cm to 3.0 cm. However, there was no significant difference in mean size between the two groups (*p* = 0.35). According to histopathologic results, malignancy accounted for more than 70%. It showed no significant difference between the two group (*p* = 0.66). Benign lesions included fibroadenoma, phyllodes tumor, and intraductal papilloma.

**Table 2:** Baseline characteristics of included patients.

	Study group N = 52	Control group N = 56	P
**Age (median, range)**	49.5 (28-70)	49 (31-75)	0.28
**Height (mean ± SD, cm)**	160.0 ± 6.1	159.2 ± 6.1	0.50
**Weight (mean ± SD, kg)**	59.9 ± 9.9	57.9 ± 9.1	0.16
**Menopausal state (%)**			
Premenopausal	27 (51.9)	39 (69.6)	0.06
Postmenopausal	25 (48.1)	17 (30.4)	
**Injection dose (mean ± SD, mL)**	0.2 ± 0.0	0.4 ± 0.1	
**Exposure period (mean ± SD, day)**	0.7 ± 0.7	0.6 ± 0.6	0.86
**Size on US (mean, range, cm)**	1.0 (0.3-2.4)	1.1 (0.1-3.0)	0.35
<1	23 (44.2)	24 (42.9)	0.27
1≤<2	27 (51.9)	25 (44.6)	
2≤	2 (3.9)	7 (12.5)	
**Pathology**			
Benign	12 (23.1)	15 (26.8)	0.66
Malignancy	40 (76.9)	41 (73.2)	

US, ultrasonography.

Negative resection rate, the primary efficacy endpoint, was 98.04% (50/51) in the study group and 90.57% (48/53) in the control group. As a result of a non-inferiority test, the lower limit of Chan and Zhang’s 95% two-sided accurate confidence interval for the difference between the two groups was -0.0231, which exceeded the non-inferiority threshold of -0.225. Thus, the accuracy of resection in the study group was not inferior to that of the control group (90.57% vs 98.04%, 95% CI: -2.31 – 18.91, p = 0.21). As a result of pathology, there was no significant difference in mean length of breast lesion or mean length of excised specimen between the two groups. However, there was a statistically significant difference in histopathological accuracy (0.26 ± 0.13 vs 0.33 ± 0.17, p = 0.01) (**Table 3**). The technical success rate was 98.0% in the study group and 88.24% in the control group for the breast. It was 100% in the study group and 92.54% in the control group for the excised specimen. Technical success rates show no statistically significant difference between the two groups (marking on breast, *p* = 0.11, marking on the excised specimen, *p* = 0.12). In the control group, skin pigmentation was observed in 16 patients, but there was no skin pigmentation case in the study group (0.00% vs. 30.77%, *p*< 0.01) (**Table 4**). Adverse events were not reported in either group.

**Table 3:** Primary endpoints.

	Study group % (n/N)	Control group % (n/N)	P
**Accuracy of resection**			
Negative resection margin	98.04 (50/51)	90.57 (48/53)	
Positive resection margin	1.96 (1/51)	9.43 (5/53)	
Difference^a^	7.47		
[95% CI^b^]	[-2.31, 18.91]		0.21
	Study group Mean ± SD	Control group Mean ± SD	P
**Histopathological accuracy**	0.26 ± 0.13	0.33 ± 0.17	0.01
The longest length of breast lesion (cm)	1.15 ± 0.56	1.36 ± 0.66	0.12
The longest length of excised specimen (cm)	4.54 ± 1.35	4.44 ± 1.35	0.70

a Difference=study group-control group.

b Chan and Zhang’s exact confidence interval.

CI, confidence interval; SD, standard deviation.

**Table 4:** Secondary endpoint.

	Study group % (n/N, 95% CI)	Control group % (n/N, 95% CI)	P
**Technical success rate**			
Marking on breast	98.0 (49/51, 89.35-99.95)	88.2 (45/53, 76.13-95.56)	0.11
Marking on excised specimen	100.0 (51/51, 93.02-100.00)	92.5 (49/53, 81.79-97.91)	0.12
**Skin pigmentation**	0 (0/50, 0.00-7.11)	30.8 (16/52, 18.72-45.10)	<0.01

CI, confidence interval.

## Discussion

Due to an increase in screening examinations and the development of imaging methods, the detection of small breast lesions is increasing. Thus, accurate localization is required for proper diagnosis and treatment (20). Among several localization techniques, needle localization has been widely used. However, it has several disadvantages, including risk of wire migration or withdrawal, patient’s pain, and interference with surgical approaches (2, 6, 7). Localization using dyes for visualizing non-palpable breast lesions is rapid and easy to perform. Among various bioavailable dyes, charcoal has been widely used as a material without the risk of fast dye dispersion. Thus, surgery can be planned several days after localization. However, it has disadvantages of skin pigmentation and foreign body reaction (9).

Because the use of ICG-HA for breast localization was not reported yet, we previously conducted a phase 2 clinical trial of ICG-HA. However, the sample size was small (n = 44). In addition, only breast benign diseases were included (18). In this study, we evaluated the efficacy and safety of ICG-HA for localizing non-palpable breast lesions including breast cancer with a larger sample size (n = 108). The accuracy of resection in the study group was not inferior to that of the control group (90.57% vs. 98.04%, 95% CI: -2.31 – 18.91, *p* = 0.21) (**Table 3**). There were significant intergroup differences in histopathological accuracy (0.26 ± 0.13 vs. 0.33 ± 0.17, *p* = 0.01) and skin pigmentation rates (0% vs. 30.8%, *p<* 0.01) (**Table 4**). However, there was no significant differences in technical success rate or adverse drug reaction.

Histopathological accuracy defined as the longest length of the breast lesion compared to the longest length of the resected specimen was evaluated to determine whether the target lesion was accurately excised without unnecessary wide resection. Similarly, in the phase 2 clinical trial, the accuracy of resection defined as the maximum diameter of the resected specimen divided by the maximum diameter of the preoperative lesion detected in the US was evaluated and the control group was found to have a higher value than the study group. The reason for the wider resection in the control group was because the injection amount of activated charcoal was higher than that of the ICG-HA (18). On the contrary, histopathological accuracy was higher in the control group than that in the study group, although the injection amount of activated charcoal was not reduced in this phase 3 clinical study. This inconsistency between the two studies might be because radical excision was intended to excise a wider area for a malignant tumor than for a benign lesion.

Skin pigmentation was observed in 30.8% of patients in the control group but in none of patients in the study group (*p*< 0.01). Skin pigmentation caused by charcoal localization can be removed by excising the overlying skin (21). However, excessive skin excision might cause poor postoperative breast shape. Most charcoal are removed during the surgery, although small amounts might remain in the breast around the surgical area. In most cases, residual charcoal does not appear on follow-up imaging. However, in some cases, residual charcoal can develop into foreign body granulomas (22). We observed only skin pigmentation as a disadvantage of charcoal in this study. Long-term follow-up is required to determine the development of foreign body granulomas that can cause unnecessary biopsy or surgery.

The technical success rate evaluated by two aspects showed a higher success rate in the study group, although the differences between the two groups was not statistically significant. In some cases of the control group, charcoal spread around the lesion with target lesion not localized. The possible cause of failure in these cases might have been the difficulty of injection into a very dense breast or hard mass. Because activated charcoal is in particulate form and insoluble in water, it usually does not disperse into the surrounding tissues, allowing surgery to be planned over several days. However, a thick needle is required for injection. Blockage of the needle tip could occur. Thus, so accurate localization is not possible because it is not gently injected into a hard tissue. On the other hand, ICG-HA has the advantages of being gently injected into hard tissues even with a thinner needle, leading to less pain for the patient.

This study had several limitations. First, although ICG is safe and the most widely used fluorophore in various clinical fields, long-term follow-up of ICG-HA has not been performed. Additional study should be conducted regarding the safety issue in the future. Second, our patient group was heterogeneous as both malignant and benign lesions were included. Therefore, there was a difference in setting an appropriate resection area for the targeted lesion. There may have been intentions of surgeons to remove wider region during surgery to obtain safer negative resection margin for cancer patients. In addition, skin pigmentation might have been underestimated because some malignant lesions required skin resection, although skin resection was not required in most benign cases. Third, since it was a multicenter study, errors might have occurred by each institution and by each researcher. Even with these limitations, this study was meaningful because it was prospectively designed and conducted on breast cancer. No studies on ICG-HA for breast localization have been reported yet.

In conclusion, this multicenter phase 3 clinical trial evaluated the efficacy and safety of an ICG-HA mixture for localization of non-palpable breast lesions relative to those of activated charcoal. ICG-HA injection is a new method for localizing non-palpable breast lesions. It is useful method for obtaining accurate resections and cosmetic benefits in breast cancer. To avoid skin pigmentation, localization with ICG-HA could be considered.

## Data availability statement

The original contributions presented in the study are included in the article/supplementary material. Further inquiries can be directed to the corresponding author.

## Ethics statement

The studies involving human participants were reviewed and approved by the ethics committee of Samsung Medical Center. The patients/participants provided their written informed consent to participate in this study. Written informed consent was obtained from the individual(s) for the publication of any identifiable images or data included in this article.

## Author contributions

SK, M-HL, SYL, and HS contributed to conception and design of the study. YB organized the database. YB and HC performed the statistical analysis. YB wrote the first draft of the manuscript. YB and HC wrote sections of the manuscript. All authors contributed to the article and approved the submitted version.
